# Detection of Protein Aggregates in Brain and Cerebrospinal Fluid Derived from Multiple Sclerosis Patients

**DOI:** 10.3389/fneur.2014.00251

**Published:** 2014-12-02

**Authors:** Monique Antoinette David, Mourad Tayebi

**Affiliations:** ^1^Mitchell Center for Alzheimer’s Disease and Related Brain Disorders, Department of Neurology, University of Texas Houston Medical School, Houston, TX, USA; ^2^Antibody Discovery Laboratory, PrioCam, Houston, TX, USA; ^3^Department of Pathology and Infectious Diseases, Faculty of Health and Medical Sciences, University of Surrey, Guildford, UK

**Keywords:** multiple sclerosis, protein-misfolding disease, soluble oligomers, monoclonal antibodies

## Abstract

Studies of the properties of soluble oligomer species of amyloidogenic proteins, derived from different proteins with little sequence homology, have indicated that they share a common structure and may share similar pathogenic mechanisms. Amyloid β, tau protein, as well as amyloid precursor protein normally associated with Alzheimer’s disease and Parkinson’s disease were found in lesions and plaques of multiple sclerosis patients. The objective of the study is to investigate whether brain and cerebrospinal fluid (CSF) samples derived from multiple sclerosis patients demonstrate the presence of soluble oligomers normally associated with protein-misfolding diseases such as Alzheimer’s disease. We have used anti-oligomer monoclonal antibodies to immunodetect soluble oligomers in CSF and brain tissues derived from multiple sclerosis patients. In this report, we describe the presence of soluble oligomers in the brain tissue and cerebral spinal fluid of multiple sclerosis patients detected with our monoclonal anti-oligomer antibodies with Western blot and Sandwich enzyme-linked immunosorbent assay (sELISA). These results might suggest that protein aggregation plays a role in multiple sclerosis pathogenesis although further and more refined studies are needed to confirm the role of soluble aggregates in multiple sclerosis.

Multiple sclerosis, the prototypical human inflammatory demyelinating disease of the central nervous system (CNS), represents a significant health burden, affecting the quality of life of many people.

Despite the important role played by T cells in the pathogenesis of MS, it is now well recognized that B cells and humoral immune responses form an important component of the mechanisms underlying disease pathogenesis (1). Oligoclonal immunoglobulins of IgG and IgM isotype are consistently recognized in all forms of MS and their status informs prognosis in MS patients (2) and are used as a diagnostic tool for the disease. These oligoclonal immunoglobulins together with memory B cells and plasma cells that led to their secretion were consistently isolated from patients with MS. Surprisingly, these memory B cells and plasma cells and the secreted immunoglobulins were demonstrated even at onset of clinical symptoms of MS, suggesting prolonged and continuous antigen stimulation likely by auto-antigens (3). The specific subtype of B cell isolated in patients with MS is recognized as part of the so-called T-independent B-cell immune response, and sequencing the immunoglobulins CDR regions derived from these B cells demonstrated abnormal pattern of somatic hypermutation (4). T-independent B-cell activation is part of the humoral immune response to pathogens; consequently, antigen alone, or antigen plus signals provided by cells other than T cells can provide all necessary signals in order to induce a B-cell response (5). Bachmann and colleagues demonstrated the importance of T-independent B-cell responses through stimulating antigen-specific B cells to proliferate and secrete IgM *in vivo* in T-cell-deficient mice (6). We have also shown previously that immunization with amyloid component of native prions leads to T-independent B-cell immune response with chronic secretion of abnormally hyper mutated IgM (Tayebi and David, Personal communication). In prion disease, failure of classical immune protection to “neutralize” auto-antigens suggests a highly aggregated state of these proteins and their ability to resist low pH in the endosomal environment renders them unable to properly process such proteins in an MHC class II-restricted pathway that involves T-cell help (7). The failure to process these auto-antigens through classical pathways has perhaps led the immune system to use “alternative” pathways, namely the T-independent B-cell responses as is well recognized with so-called thymus-independent (TI)-1 and 2 antigens (6).

Amyloid deposits are described in many CNS disorders. In Alzheimer’s disease (AD), the commonest form of human amyloidosis, amyloid deposits are displayed as extra-cellular senile plaques in the gray matter and cortex. Amyloid plaque formation is also seen in gray matter of brains that are prion diseased.

In MS, amyloid precursor protein (APP), abundantly found in amyloid plaques associated with AD pathogenesis (8), was associated with lesions and plaques (9). Furthermore, patients with primary progressive MS displayed increase of amyloid β (Aβ) and tau protein levels in their CSF.

In this study, we investigated whether soluble oligomers, common to most amyloids, may be present in CSF and brain tissues of MS patients to lay the ground for further studies characterizing the toxicity of soluble oligomeric species and its involvement in MS pathogenesis. We hypothesized that oligoclonal antibodies consistently found in MS patients (10) are stimulated by toxic soluble oligomers and are secreted through the so-called T-cell-independent B-cell immune response.

We have recently demonstrated that monoclonal antibodies (mAbs) called PRIOC mAbs raised against prion oligomers were also able to immunodetect synthetic Aβ and α-synuclein soluble oligomers (11). The PRIOC mAbs were produced following subcutaneous immunization of FVB/N *Prn-p^0/0^* with Dynabead-adsorbed prions. PrP^C^ or PrP^Sc^-positive hybridomas were generated for the production of mAbs specific for soluble oligomers. These PRIOC mAbs were shown to bind only the oligomeric species but failed to bind to both the monomers and the fibril counterparts. Similarly, our PRIOC mAbs were also able to bind synthetic oligomeric species of tau and islet amyloid polypeptide (IAPP) associated with tauopathies and type 2 diabetes, respectively, but not with their monomeric and fibrils counterparts (data not shown). Kayed and colleagues (12) have also shown that polyclonal sera raised against Aβ peptide oligomers also detected α-synuclein, IAPP, polyglutamine, lysozyme, human insulin, and prion oligomers. Taken together, these results argue that a conformational epitope is detected by PRIOCs, independently of their primary sequences. We then tested the ability of the PRIOC mAbs to detect Aβ soluble oligomers in AD patients by immunohistochemistry and immunofluorescence as previously described (11, 13). Here, we show that PRIOC antibody staining displayed a diffuse pattern of medium-sized intracellular aggregates, consistent with staining of soluble oligomers (Figure 1). In contrast, the Aβ-4G8 (14) and the AT8-tau (15) specific antibody bound large extra-cellular Aβ plaques and intracellular tau aggregates, respectively (Figure 1).

**Figure 1:**
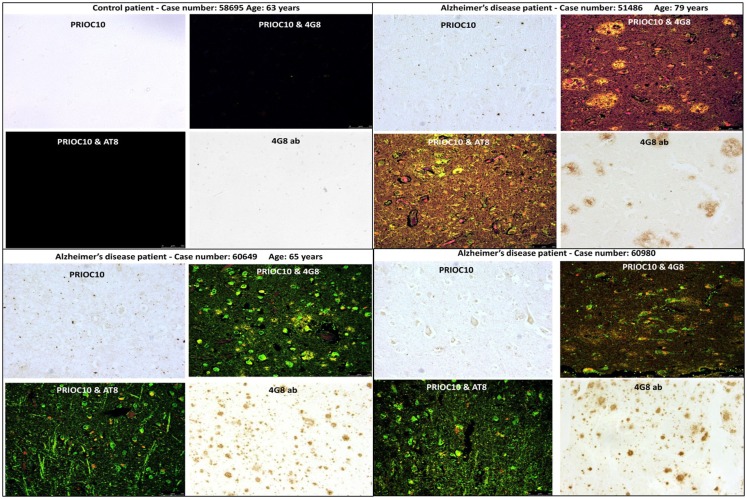
**Immunodetection of Aβ and tau oligomers in brains of Alzheimer’s disease patients with the PRIOC monoclonal antibodies**. Brain sections (7 μm thickness) derived from Alzheimer’s disease patients (patient case number 51486; 60649; 60980) and from a control brain derived from a patient with cancer (patient case number 58695) were stained with PRIOC, 4G8 (stains Aβ plaques), or AT8 mAb (stains phosphorylated tau). The control 58695 case displayed no staining for oligomers, Aβ plaques, and pathological tau. The 51486 case showed a diffuse pattern of medium-sized aggregates with PRIOC mAb and large plaques with 4G8. Confocal studies displayed co-staining of both the plaques and the aggregates. Cases 60649 and 60980 displayed extensive intracellular staining (green fluorescence) and smaller plaques.

These antibodies were used to detect soluble oligomers in brain homogenates and CSF from patient with MS; surprisingly, the anti-oligomer mAbs were able to specifically recognize oligomers by Western blot in four patients with MS but not in controls. Our anti-oligomer mAbs detected synthetic oligomers produced from monomeric recombinant PrP (rPrP), Aβ peptide, and α-synuclein (11). We showed that only the oligomeric species derived from monomers were immunodetected by PRIOC mAbs that failed to bind to both the monomers and the fibrils (11).

In this study, we investigated whether soluble oligomers, common to most amyloids, may be present in CSF and brain tissues of MS patients to lay the ground for further studies characterizing the toxicity of soluble oligomeric species and its involvement in MS pathogenesis. Fresh frozen MS and normal control brain samples obtained at autopsy under The UK Multiple Sclerosis Tissue Bank-approved protocol (Table 1) were used in this study. Samples derived from cerebral hemispheres were harvested then frozen at −80° C until further use. CSF and sera from MS, AD, Parkinson’s disease (PD), and control patients provided by The UK Multiple Sclerosis Tissue Bank were also used.

**Table 1:** **Summary details of MS cases used in this preliminary study**.

Case number	Type	Died age (years)	Sex	Brain weight (grams)	CSF pH	Cause of death	Death-tissue preservation interval (hours)	Tissue provided	Preservation protocol
MS050*	SPMS	72	F	1175	–	BP; MS	8	B; SC	P; FF; SF
MS058*	SPMS	51	F	1000	–	MS	15	B; SC	P; FF; SF
MS062*	SPMS	49	F	935	–	RI	10	B; SC	P; FF; SF
MS074*	SPMS	64	F	889	7.42	AP	7	B; SC; CSF	P; FF; SF
MS076*	SPMS	49	F	1139	6.24	CRF; HD	31	B; SC; CSF	P; FF; SF
MS079*	SPMS	49	F	969	6.94	BP; MS	7	B; SC; CSF	P; FF; SF
MS094*	PPMS	42	F	1229	–	BP; MS	11	B; CSF	P; FF; SF
MS097*	SPMS	55	M	1190	–	BP; MS	31	B; SC; CSF	P; FF; SF
MS122*	SPMS	44	M	1400	6.19	BP	16	B; SC; CSF	P; FF; SF
MS166*	SPMS	52	F	891	–	BP; MS	7	B	P; FF; SF
MS200*	SPMS	44	F	1205	6.9	UTI; S; MS	20	B; SC	P; FF; SF
MS229*	SPMS	53	M	1219	7.2	BP; MS	13	B; SC	P; FF; SF
MS302*	SPMS	59	M	995	6.5	S; UTI	8	B; SC	P; FF; SF
MS311*	SPMS	45	F	973	–	P	22	B; SC	P; FF; SF
MS313*	PPMS	66	M	1266	7.32	PU; MS	16	B; SC	P; FF; SF
MS060*	SPMS	55	M	1360	6.58	MS	16	CSF	SF
MS082*	SPMS	49	F	1057	–	AP	9	CSF	SF
MS154*	SPMS	34	F	1244	7.5	P	12	CSF	SF
MS104*	SPMS	53	M	1100	6.55	MS	12	CSF	P; FF; SF
MS106*	NS	39	F	1010	6.48	BP	18	CSF	SF
CO41*	control	54	M	1829	7.5	LC	20	B; SC; CSF	P; FF; SF
PD020*	PD	75	M	1351	–	–	12	B; SC; CSF	P; FF; SF
MD01	PPMS	–	–	–	–	–	–	CSF; Blood	SF
MD02	SPMS	–	–	–	–	–	–	CSF; Blood	SF
58695	C	63	M	–	–	Cancer	–	B	P; FF
51486	AD	79	M	–	–	AD	–	B	P; FF
60649	AD	65	M	–	–	AD	–	B	P; FF
60980	AD	–	M	–	–	AD	–	B	P; FF

PPMS, primary progressive; SPMS, secondary progressive; PD; Parkinson’s disease; ND, not done; NS, not specified; BP, bronchopneumonia; AP, aspiration pneumonia; P, pneumonia; LC, lung cancer; RI, respiratory infection; CRF, chronic renal failure; HD, heart disease; UTI, urinary tract infection; S, septicemia; PU, peptic ulcer; NK, not known; B, brain; SC, spinal corf; CSF, cerebrospinal fluid; P, paraffin; FF, fixed-frozen; SP, snap-frozen. *Provided by The UK Multiple Sclerosis Tissue Bank.

We initially tested two CSF samples from MS patients (MD01 and MD02) for the presence of immunoglobulins using autologous serum from these patients (Figure 2). Following centrifugation of 2 ml of CSF aliquots (800 × *g*, 5 min), supernatants were kept at −80°C until further analyses. Trichloroacetic acid (TCA)/acetone precipitation protocol was used to precipitate proteins from supernatants and total protein concentrations were measured using the BCA Protein Assay (Pierce). We reasoned that serum of patients with MS would contain specific IgG and/or IgM antibodies that can discriminate against proteins (oligomers and others) associated with the pathologic process. In that context, 20 μl of 10 mg/ml total protein derived from CSF samples (primary progressive: CSF 1 and secondary progressive: CSF 2) from MS patients were electrophoresed by Western blot then probed with serum (serum 1 and serum 2) from the same patients. Anti-IgG or anti-IgM HRP conjugated antibody (1 in 10,000) was used as secondary detection antibody. Sera from both MS patients were able to immunodetect an extra band (~95 kDa) in CSF from the same patients (Figure 2). Of note, the secondary antibodies were unable to bind to the 95 kDa band when sera were omitted, confirming its disease specificity. Finally, the 95 kDa band was recognized by both IgG and IgM antibody present in sera of MS patients (Figure 2).

**Figure 2:**
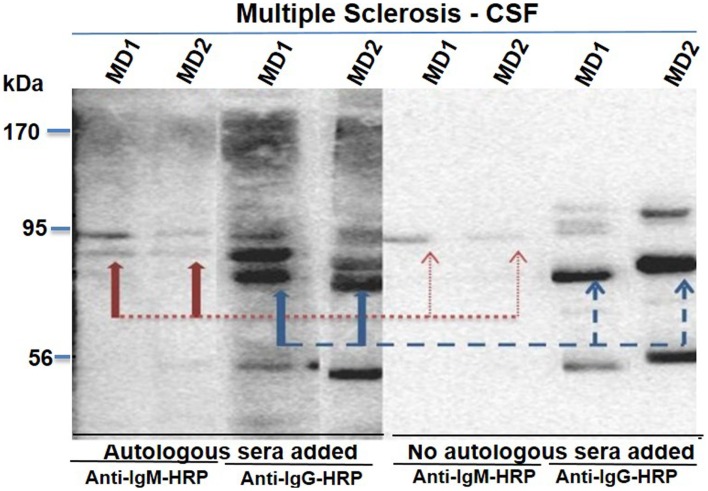
**Presence of oligoclonal bands in CSF samples from MS patients**. CSF samples from MS (primary progressive: MD01 and secondary progressive: MD02) were electrophoresed then probed with autologous serum (1, 2, 3, and 4). Secondary anti-IgM (1 and 2) or anti-IgG (3 and 4) HRP-conjugate was used for detection of the autologous sera. Sera were omitted from lanes 5, 6, 7, and 8 to confirm their binding specificity after addition of secondary anti-IgM (5 and 6) or anti-IgG (7 and 8) HRP-conjugate.

Next, we set out to determine whether soluble oligomers would be detected by anti-oligomer PRIOC mAbs in brains and CSF of MS patients. Brain samples (MS074, MS076, MS079, and MS094) to be electrophoresed were diluted 1:1 in 40 ml sample buffer and boiled for 5 min in eppendorf tubes. The samples were spun for 5 s at 14,000 rpm in a microfuge before being loaded on the gel. CSF samples were prepared as described above. We probed for soluble oligomers with Western blot and we show for the first time that our anti-oligomer mAbs were able to detect soluble oligomers in human brain homogenates and CSF from MS patient but not in control brain (Figure 3A). Furthermore, Western blot analysis of human brain homogenates and CSF from MS patients revealed that our mAbs were able to detect several bands ranging between 90 and 200 kDa (Figure 3A), consistent with the smeary appearance normally seen with similar antibodies used by other researchers to immunodetect soluble oligomers. Control brain isolated from a young patient who died from lung cancer (Ct, CO41) did not display soluble oligomers-specific bands on Western blot (Figure 3A); in contrast and as expected, Western blot of brain and CSF isolated from a patient with Parkinson’s disease (PD; PD020) led to detection of soluble oligomers (Figure 3A). We then expanded our study to include 27 MS samples to confirm the presence of the soluble oligomers in brain and CSF and validate the above data. We used our previously published Sandwich ELISA assay specifically developed to immunodetect soluble oligomers (11). The assay relies on a two-site system that includes an immunocapture PRIOC antibody with the ability to capture the oligomers, followed by Immunodetection with biotinylated PRIOC. All 15 brain homogenates derived from MS patients (Table 1) displayed high levels of soluble oligomers by ELISA, similar to levels seen in AD and PD patients but not in the cancer patients (Figure 3B). Twelve CSF samples derived from the MS patients (Table 1) also showed high OD values by ELISA albeit these were a third to half the levels seen in the brain homogenates (Figure 3B).

**Figure 3:**
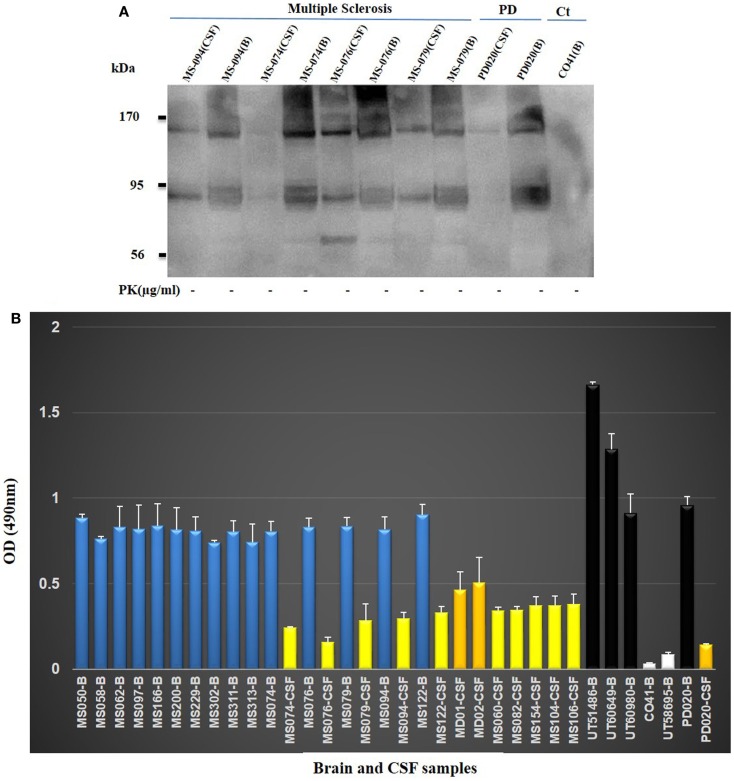
**(A) Detection of soluble oligomers in brain homogenate and CSF of patients with multiple sclerosis**. Brain homogenates and CSF samples MS094; MS074; MS076; MS079) from MS patients were probed with anti-oligomer mAbs. Several bands (90–200 kDa range) consistent with the oligomeric profile recognized on Western blotting have been detected. Positive control Parkinson’s disease (PD) brain homogenate and CSF displayed similar pattern as opposed to the human control (Ct, CO41) brain, which failed to show oligomer detection. **(B)** Brain homogenates and/or CSF derived from MS, PD, AD, and lung cancer were used to assess specific binding of PRIOC mAb to soluble oligomers in a Sandwich ELISA assay. PRIOC was used to coat the ELISA plate in coating buffer. Brain homogenate and/or CSF were added to the wells followed by a biotinylated PRIOC mAb. Error bars represent the mean antibody level derived from *n* = 4 wells.

Proteinase K (PK)-sensitive oligomers are considered the toxic species in protein-misfolding diseases (PMDs) and have been demonstrated by many research groups (7). We wanted to confirm whether soluble oligomers immunodetected in MS displayed resistance to PK and behaved in a prion-like fashion. Brain homogenates from patients with MS (secondary progressive) and PD were subjected to appropriate concentration of PK; as low as 6.25 μg/ml of PK led to complete digestion of the soluble oligomers (Figure 4A), consistent with previous work with PD and prion.

**Figure 4:**
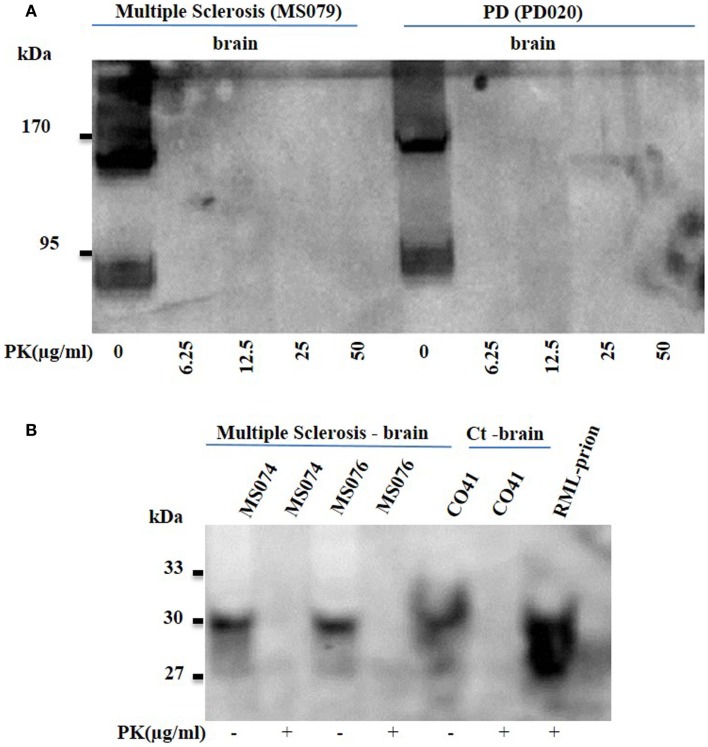
**(A)** Proteinase K sensitive oligomers or prions in brain homogenate of patients with multiple sclerosis. Brain homogenates from MS patients (MS079) were probed with anti-oligomer mAbs. The bands (90–200 kDa range) demonstrated with the anti-oligomer antibody in both MS and the positive control Parkinson’s disease (PD) brain homogenates via western blotting, were no longer seen following proteinase K digestion. **(B)** Brain homogenates (MS074; MS076) from MS patients and the human control (Ct; CO41) brain were probed with anti-PrP 3F4 mAb to immunodetect the typical PrP bands. RML (PrP^Sc^)-infected brain homogenate was used to probe for the PK sensitivity and was shown to display the 27–30 kDa band.

Finally, co-morbidities are a recognized feature following autopsy of patients with PMDs; for instance, both Parkinson’s and AD have been recognized in same patients. We therefore wanted to assess whether brain homogenates derived from MS patients displayed PK-resistant prions (Figure 4B) and we also checked for the presence of Aβ and α-synuclein (data not shown). Brain homogenates from patients with MS (Secondary progressive) did not display any presence of PK-resistant prions as assessed by anti-prion antibody and also failed to demonstrate binding for Aβ and α-synuclein.

Our study shows for the time that protein aggregates detected by anti-oligomer specific antibodies are associated with MS. The data generated so far does not allow to reach a substantive conclusion in relation to the involvement of these proteins aggregates in MS pathogenesis. Protein aggregation associated with MS has been described previously in a rodent model of MS (16). Dasgupta and colleagues have demonstrated increased protein aggregation in the spinal cord of mice with experimental autoimmune encephalomyelitis (EAE). In their study, they argue that accumulation of misfolded proteins is caused by increased rate of protein oxidation and reduced proteasome degradation, both seen in MS (17) and EAE (18, 19).

In conclusion, for the first time, we demonstrate the presence of soluble oligomers, normally associated with PMDs, in MS tissues and CSF. Although the pathogenic relevance of these MS associated soluble oligomers to disease process remains to be investigated, this study sets the ground for further investigating this relationship. This study proposes a novel alternative in understanding the pathogenesis of MS.

## Author Contributions

Monique Antoinette David designed and performed the experiments; Mourad Tayebi designed and managed experiments and wrote the manuscript.

## Conflict of Interest Statement

The authors declare that the research was conducted in the absence of any commercial or financial relationships that could be construed as a potential conflict of interest.
